# The Spectrum of Heart Failure Management

**DOI:** 10.7759/cureus.40587

**Published:** 2023-06-18

**Authors:** Usman Ghani, Omer Farooq, Sundal Aziz, Sundus Alam, Muhammad Junaid Khan, Omar Rahim

**Affiliations:** 1 Cardiology, Northwest General Hospital and Research Center, Peshawar, PAK; 2 Internal Medicine, Ascension Saint Francis Hospital, Evanston, USA; 3 Acute Medicine, Gloucestershire Hospitals NHS Foundation Trust, Gloucester, GBR; 4 Orthopaedic Surgery, Gloucestershire Hospitals NHS Foundation Trust, Gloucester, GBR; 5 Cardiology, Naseer Teaching Hospital, Peshawar, PAK

**Keywords:** regenerative medicine, transplant, device therapy, medication, lifestyle, monitoring, classification, management, heart failure

## Abstract

Heart failure, a complex cardiovascular condition, is a huge burden on patients, caregivers, and healthcare systems and it is prevalent worldwide. Heart failure is caused by a wide variety of underlying conditions that include both cardiac and non-cardiac pathologies. Identifying the underlying cause enables us to apply etiology-based interventions. The spectrum of heart failure management ranges from classification to transplantation. In addition to its classification and monitoring, this article reviews various management strategies, including both conventional methods and the latest innovations. These include lifestyle interventions, pharmacotherapy, device therapy, transplantation, and regenerative medicine.

## Introduction and background

Heart failure occurs when the heart is unable to pump enough blood to fit the body’s requirements. Classification systems for this condition have been provided by the New York Heart Association and the American College of Cardiology/American Heart Association. These classifications can be used to evaluate the functional status and to stage heart failure. Treatment strategies can then be tailored. The goal of these treatments is to reduce associated morbidity and mortality.

Heart failure is caused by a variety of conditions, and identifying the underlying cause helps us to optimize treatment. It is also imperative to monitor the disease. In this review, we discuss various evaluation and monitoring strategies that form the core elements of managing heart failure. Signs of disease progression and early decompensation detected on monitoring necessitate modifications in therapeutic interventions to reduce morbidity and mortality.

To manage heart failure, it is essential to adopt a multifaceted approach. Conventional methods of managing heart failure include lifestyle changes and pharmacotherapy, whereas, interventional heart failure therapy utilizes minimally invasive procedures to improve outcomes [1]. Cardiac transplantation, the gold standard treatment of heart failure [2], is usually the last resort and is recommended when other strategies have failed to provide sufficient benefit. Regenerative medicine and novel pharmacological agents are promising avenues for research and could potentially be the future of heart failure management.

## Review

Methods

A comprehensive review of the literature related to heart failure management was conducted. Data were collected from electronic databases (PubMed and Scopus) to identify relevant peer-reviewed articles. The following search terms were used: “heart failure’’, “management”, “classification”, “monitoring”, “lifestyle”, “medication”, “device therapy”, “transplant” and “regenerative medicine”. We included articles in the English language only. The inclusion criteria were not exclusive to any specific study design, and 288 relevant articles were included in the review. Findings were presented using a narrative approach. The data were summarized to highlight the classification, causes, and management options.

Classification of heart failure

The most common classification system is the New York Heart Association classification system. This classification categorizes patients into four classes based on symptoms and limitations of activity. Class I and II are considered mild heart failure, whereas, class III and IV are considered severe/advanced heart failure. These four levels are presented in Table 1.

**Table 1:** New York Heart Association functional classification of HF* *[3] HF: heart failure

Class	Patient symptoms
Class I	No limitation in physical activity. Ordinary physical activity does not cause symptoms of HF
Class II	Slight limitations in physical activity. Comfortable at rest but ordinary physical activity results in symptoms of HF
Class III	Marked limitation in physical activity. Comfortable at rest but less-than-ordinary activity causes symptoms of HF
Class IV	Unable to carry on any physical activity without symptoms of HF; symptoms of HF even while at rest

The American College of Cardiology/American Heart Association staging system includes four stages (Table 2).

**Table 2:** The American College of Cardiology/American Heart Association stages of heart failure* *[3] HF: heart failure

Stage	Objective assessment
Stage A	At risk for HF but without structural heart disease or symptoms of HF
Stage B	Structural heart disease but without signs or symptoms of HF
Stage C	Structural heart disease with prior or current symptoms of HF
Stage D	Refractory HF requiring specialized interventions

According to the latest guidelines, heart failure is classified based on the ejection fraction into the following classes. In heart failure with reduced ejection fraction, the left ventricular ejection fraction is ≤40%. If the ejection fraction improves later on, it is then called heart failure with improved ejection fraction. In heart failure with mildly reduced ejection fraction, the left ventricular ejection fraction ranges from 41% to 49%. In heart failure with preserved ejection fraction, the left ventricular ejection fraction is ≥50% [4].

Causes

Over two-thirds of the total cases of heart failure can be attributed to ischemic heart disease, hypertensive heart disease, chronic obstructive pulmonary disease, and rheumatic heart disease [5]. Ischemic heart disease remains the leading cause, accounting for 26.5% of the age-standardized prevalence rate, followed closely by hypertensive heart disease, accounting for 26.2%, and chronic obstructive pulmonary disease, accounting for 23.4% [6]. Other causes include cardiomyopathies, arrhythmias, congenital heart disease, myocarditis, endocarditis, non-rheumatic degenerative mitral valve disease, non-rheumatic calcific aortic valve disease, Chagas disease, interstitial lung disease, emphysema, pulmonary hypertension, pulmonary embolism, thyrotoxicosis, immune disorders, obesity, diabetes, and sleep apnea.

The causes of heart failure vary between developing and developed countries. The underlying causes in developed countries are usually ischemic heart disease and chronic obstructive pulmonary disease, whereas, in developing countries, hypertensive heart disease, cardiomyopathies, myocarditis, and rheumatic heart disease are prevalent [5]. The INTERHEART risk score was highest in developed countries (p<0.001), indicating the greatest risk factor burden. However, the rates of major diseases and death were substantially lower in developed countries compared to those in developing countries. These improved outcomes in developed countries, despite a greater risk factor burden, may be attributed to better healthcare provision. The use of pharmacotherapy and revascularization is significantly more common in developed countries (p<0.001) [7].

Evaluation and monitoring

Diagnosing heart failure requires examining both clinical features and objective findings.

History and Examination

The evaluation should start with a thorough history and physical examination. The symptoms of heart failure include orthopnea, paroxysmal nocturnal dyspnea, lethargy, fatigue, reduced exercise tolerance, nocturnal cough, wheezing, and ankle swelling. The signs are displaced apex beat, right ventricular heave, third heart sound, tachycardia, pulsus alternans, elevated jugular venous pressure, wheezing, crepitations, cachexia, muscle wasting, edema, hepatomegaly and ascites [8]. Daily weight monitoring is also frequently advised [9].

Electrocardiography

Electrocardiographic abnormalities are common in suspected cases; therefore, an abnormal test has very little predictive value. On the other hand, if the electrocardiography findings are normal, an alternative diagnosis should be carefully considered [10].

Biomarkers

Testing for cardiac biomarkers is commonly carried out as they are a cost-effective means of ruling out heart failure. Cardiac biomarkers associated with myocardial stretch are brain or b-type natriuretic peptide (BNP), n-terminal pro-b-type natriuretic peptide (NT-proBNP), and mid-regional pro atrial natriuretic peptide (MR-proANP). Biomarkers associated with myocyte injury are high-sensitivity troponin T (hsTnT) and heart-type fatty acid-binding protein (H-FABP). Biomarkers associated with fibrosis, inflammation, and matrix remodeling are soluble growth-stimulating gene 2 (sST2), galectin-3 (Gal-3), and growth differentiation factor-15 (GDF-15) [11]. Among all these markers, the ones commonly used in clinical practice are BNP and NT-proBNP.

Imaging

Chest X-ray can identify cardiomegaly and pulmonary congestion. Using transthoracic Doppler echocardiography, we can measure the left ventricular ejection fraction. This enables us to determine whether the systolic function is preserved or reduced. Fast breath-hold cardiovascular magnetic resonance (CMR) is the reference standard for the measurement of cardiac volumes, function, and mass in heart failure. The results are accurate and reproducible [12].

Remote Monitoring

Remote monitoring can either be invasive or non-invasive.

Non-invasive remote monitoring is simple and cost-effective. It enables consistent monitoring of blood pressure, physical activity, and weight. It also improves medication adherence [13]. Some studies report fewer readmission rates [13], whereas, others have failed to demonstrate reduced readmission rates [14]. Wearable health devices have many potential benefits; however, the available data is limited to observational studies and small, randomized controlled trials [15].

Alternatively, remote hemodynamic monitoring requires an invasive procedure and may not be cost-effective. It has shown the strongest ability to reduce heart failure readmissions and is currently approved for this purpose [16].

Lifestyle

Diet

The American College of Cardiology/American Heart Association recommendations include restricting salt intake to less than 2 grams per day and fluid intake to less than 2 liters per day [17].

Alcohol Consumption

Although light/moderate consumption of alcohol may confer some cardiovascular benefits, excessive drinking should be avoided as it is known to cause alcoholic cardiomyopathy [18].

Smoking

Cigarette smoking is an important risk factor for left ventricular hypertrophy, systolic dysfunction, and incident heart failure hospitalization and is associated with higher mean BNP levels (p<0.05). Current smoking (hazard ratio: 2.82; 95% confidence interval: 1.71-4.64), smoking intensity among current smokers (greater than 19 cigarettes per day: hazard ratio: 3.48; 95% confidence interval: 1.65-7.32), and smoking burden among ever smokers (greater than 14 packs per year: hazard ratio: 2.06; 95% confidence interval: 1.29-3.3) were significantly associated with incident heart failure hospitalization in comparison with never smoking [19].

Weight Management

Treatment of heart failure also includes weight reduction in obese patients. Sudden unexpected weight gain of greater than 2 kilograms in three days should either be discussed with a healthcare provider or patients can adjust their diuretic dose accordingly (increase the dose if a sustained increase in weight is noted) [10]. On the other hand, malnutrition/cardiac cachexia is present in half of patients with severe congestive heart failure. The cachectic state is associated with increased mortality in these patients [20].

Activity

Although destabilization of congestive heart failure necessitates bed rest, patients are otherwise encouraged to carry out activities that do not induce symptoms.

Pharmacotherapy

Table 3 lays out the American Heart Association/American College of Cardiology recommendations for managing heart failure with reduced ejection fraction.

**Table 3:** Recommendations for managing heart failure with reduced ejection fraction by the American Heart Association/American College of Cardiology* *[4] HFpEF: heart failure with preserved ejection fraction; SGLT2i: sodium-glucose cotransporter 2 inhibitors; HF: heart failure; AF: atrial fibrillation; MRA: mineralocorticoid receptor antagonists; LVEF: left ventricular ejection fraction; ARB: angiotensin receptor blockers; ARNi: angiotensin receptor/neprilysin inhibitors

Class of recommendation	Recommendations
1	Patients with HFpEF and hypertension should have medication titrated to attain blood pressure targets in accordance with published clinical practice guidelines to prevent morbidity
2a	In patients with HFpEF, SGLT2i can be beneficial in decreasing HF hospitalizations and cardiovascular mortality
2a	In patients with HFpEF, management of AF can be useful to improve symptoms
2b	In selected patients with HFpEF, MRAs may be considered to decrease hospitalizations, particularly among patients with LVEF on the lower end of the spectrum
2b	In selected patients with HFpEF, the use of ARB may be considered to decrease hospitalizations, particularly among patients with LVEF on the lower end of the spectrum
2b	In selected patients with HFpEF, ARNi may be considered to decrease hospitalizations, particularly among patients with LVEF on the lower end of the spectrum
3 (no benefit)	In patients with HFpEF, routine use of nitrates or phosphodiesterase-5 inhibitors to increase activity or quality of life is ineffective

Angiotensin-Converting Enzyme Inhibitors

Angiotensin-converting enzyme inhibitors are used as first-line therapy in patients with reduced left ventricular ejection fraction. They inhibit the conversion of angiotensin I to angiotensin II. By reducing vasoconstriction and fluid retention, they decrease the workload on the heart. They improve symptoms, functional capacity, and survival while reducing hospitalization. The commonly used drugs are enalapril, lisinopril, and ramipril. The common side effects include cough, hypotension, renal insufficiency, hyperkalemia, syncope, and angioedema. Angioedema and bilateral renal artery stenosis are contraindications to the use of angiotensin-converting enzyme inhibitors. Enalapril is also recommended in patients with asymptomatic left ventricular dysfunction [21].

Angiotensin Receptor Blockers

Alternatively, angiotensin receptor blockers (losartan, valsartan, and candesartan) can also be used. They are particularly beneficial in patients who cannot tolerate the side effects (cough, angioedema) of angiotensin-converting enzyme inhibitors. They improve symptoms and reduce hospitalizations. They also reduce mortality, similar to angiotensin-converting enzyme inhibitors [22].

Diuretics

In patients with symptomatic heart failure, diuretics (furosemide, hydrochlorothiazide) are recommended. Diuretics increase urine production and relieve congestion. They rapidly improve dyspnea and increase exercise tolerance [23]. Loop diuretics are used for acute heart failure, whereas thiazides are used for chronic management. Further studies need to be conducted to evaluate their impact on morbidity and mortality. 

Spironolactone, an aldosterone antagonist, effectively reduces morbidity and mortality [24]. It is indicated for patients with the New York Heart Association class II-IV heart failure with reduced ejection fraction who have a creatinine clearance of greater than 30 mL per minute and serum potassium level less than 5 mEq/L. It may also reduce hospitalizations in selective patients with heart failure with preserved ejection fraction. However, hyperkalemia is a concerning side effect.

Beta-Blockers

Beta-blockers reduce heart rate, decrease myocardial oxygen demand and improve cardiac function. They are recommended for all patients with heart failure from cardiomyopathies and reduced left ventricular ejection fraction who are on pharmacotherapy unless contraindicated. They reduce morbidity and mortality [25].

Digoxin

Digoxin improves ventricular function and symptoms but does not reduce mortality. It is used as a second-line drug for mild to moderate heart failure management.

Angiotensin Receptor-Neprilysin Inhibitors

Angiotensin receptor-neprilysin inhibitors have increasingly gained popularity in the management of heart failure. In a double-blind trial, the angiotensin receptor-neprilysin inhibitor LCZ696 was proven superior to enalapril in reducing morbidity and mortality in patients with heart failure with reduced ejection fraction [26].

Sodium-Glucose Co-Transporter 2 (SGLT2) Inhibitors

Similarly, in another double-blind trial, sodium-glucose co-transporter 2 (SGLT2) inhibitors were proven to reduce morbidity and mortality (regardless of the presence of diabetes) [27].

Interventional heart failure therapy

Patients on the above-mentioned therapies with refractory symptoms are usually treated with additional invasive procedures. Device therapy is not meant to replace pharmacotherapy, but rather complement it. There has been a surge in the use of several device-based therapies for managing heart failure. The most commonly used devices are described below.

Cardiac Resynchronization Therapy

The cardiac resynchronization therapy device, also known as a biventricular pacemaker, has leads that are positioned in the right atrium, right ventricle, and coronary sinus vein on the left side of the heart. Rhythmic generation of electrical impulses synchronizes cardiac contractions and improves cardiac function. It is particularly useful in patients with heart failure and electrical dyssynchrony. This device improves exercise tolerance, quality of life, New York Heart Association class, and left ventricular ejection fraction [28].

Implantable Cardioverter-Defibrillator

The implantable cardioverter-defibrillator is useful for treating ventricular tachycardia and ventricular fibrillation. This device, implanted under the skin, detects an abnormal cardiac rhythm and delivers electrical shocks to restore sinus rhythm. Implantation of implantable cardioverter-defibrillator in addition to cardiac-resynchronization therapy with a pacemaker should be considered in symptomatic patients with severe heart failure with left ventricular ejection fraction ≤35% and QRS duration ≥120 milliseconds, as this strategy reduces morbidity and mortality [29].

Intra-aortic balloon pump and extracorporeal membrane oxygenation are mechanical support systems that can be used as a temporary bridge to cardiac transplantation.

Intra-Aortic Balloon Pump

Intra-aortic balloon pump places an inflatable balloon through the femoral artery into the aorta using a catheter. Once placed, the balloon inflates and deflates in synchronization with the cardiac cycle. It improves cardiac output via inflation and reduces afterload via deflation.

Extracorporeal Membrane Oxygenation

Extracorporeal membrane oxygenation is a cardiopulmonary support system that takes blood out of the body, replaces the carbon dioxide with oxygen, then pumps the oxygenated blood back into the body. The veno-arterial (VA) extracorporeal membrane oxygenation configuration is used in patients with heart failure. It functions similarly to a heart-lung machine (bypass used in cardiac surgeries). The major difference is that, unlike the heart-lung machine, it can be used for longer periods. Extracorporeal membrane oxygenation and intra-aortic balloon pump may have synergistic effects in the management of acute heart failure [30].

Ventricular Assist Devices

Ventricular assist devices are electromechanical devices that assist in cardiac circulation. They can partially or completely replace the function of the heart. Ventricular assist devices can increase exercise capacity, and improve symptoms and overall quality of life. The most commonly used device is the left ventricular assist device, which improves left ventricular function. In some cases, where there is significant impairment of right ventricular function as well, biventricular assist devices are used. Ventricular assist devices can be used as a bridge or alternative to cardiac transplantation [31]. Complications include thrombosis, bleeding, infection, and device malfunction.

Heart Transplant

Heart transplant is performed in patients with advanced heart failure who have failed to respond to other treatment strategies. It can significantly increase survival and exercise capacity and improve the recipient’s quality of life. The sparse availability of donor hearts is a limitation to this treatment option. The median survival of adult recipients is 10.7 years. Factors associated with early mortality are graft failure and multiple organ dysfunction. Factors associated with late mortality are malignancy, infection, rejection, and cardiac allograft vasculopathy [32].

Regenerative medicine

Stem Cell Therapy

Only a small proportion of patients with ventricular assist devices have demonstrated reverse structural remodeling of the myocardium with significant functional improvement. As a result, cell therapy for heart failure has gained popularity due to its potential to induce reverse remodeling of the myocardium [33]. Stem cells can be derived from adults and embryos. They have the capacity to differentiate into various types of cells, including cardiac cells, and regenerate damaged cardiac tissue. Certain challenges like ensuring cell survival while reducing tumorogenicity rates necessitate further studies.

Gene Therapy

Through this approach, cardiac tissue repair can be induced through the introduction of therapeutic genes (genes encoding growth factors and anti-apoptotic factors) and proteins. It has certain similar challenges as stem cell therapy (including the need to optimize delivery methods). Viral vectors (adeno-associated viruses) are usually used; however, immune response to the vector is a major concern. Some methods for transducing the cells such as the retrograde delivery approach and using cardiac-specific adeno-associated virus vectors are promising [34].

Tissue Engineering

Tissue engineering refers to constructing cardiac tissue in vitro. This tissue can then be transplanted into the heart to restore damaged areas, promote regeneration, and improve cardiac function. One approach is to seed cells onto porous, biodegradable scaffolds. Another approach is to cast cells into synthetic hydrogels or create scaffold-free tissues composed exclusively of the cells and matrix secreted by them [35]. Although early studies for tissue engineering approaches are promising, they need to be studied extensively. Common concerns include the release of toxic degradation products and graft rejection.

## Conclusions

Heart failure is a significant burden with various underlying etiologies. Monitoring enables early detection of progression, which necessitates adjustments in therapeutic interventions. Managing heart failure requires a multifaceted approach. Lifestyle changes and pharmacotherapy play a vital role in its management, whereas interventional heart failure therapy utilizes minimally invasive procedures to improve outcomes in advanced and refractory cases. Cardiac transplantation remains the gold standard, and it is employed when all other options have been exhausted. Advancements in novel pharmacological agents and regenerative medicine are promising. However, further studies need to be conducted.
